# Impact of a Thin Sacrificial Mo Layer on the Formation of the Wide Band Gap ACIGSe Absorber/ITO Thin-Film Solar Cell Interface

**DOI:** 10.1021/acsami.5c02315

**Published:** 2025-05-20

**Authors:** Angelika Demling, Rico Gutzler, Cristiana Filipa Almeida Alves, Regan G. Wilks, Roberto Félix, Dimitrios Hariskos, Stefan Paetel, Rafael Cerqueira, Sascha Sadewasser, Wolfram Witte, Marcus Bär

**Affiliations:** † Department of Interface Design, 545117 Helmholtz-Zentrum Berlin für Materialien und Energie GmbH (HZB), Berlin 12489, Germany; ‡ 54355 Zentrum für Sonnenenergie- und Wasserstoff-Forschung Baden-Württemberg (ZSW), Stuttgart 70563, Germany; § 246702 International Iberian Nanotechnology Laboratory (INL), Braga 4715-330, Portugal; ∥ Energy Materials In Situ Laboratory Berlin (EMIL), HZB, Berlin 12489, Germany; ⊥ Department of X-ray Spectroscopy at Interfaces of Thin Films, Helmholtz-Institute Erlangen-Nürnberg for Renewable Energy (HI ERN), Berlin 12489, Germany; # Department of Chemistry and Pharmacy, Friedrich-Alexander-Universität Erlangen-Nürnberg (FAU), Erlangen 91058, Germany

**Keywords:** CIGS, wide band gap, GaO_*x*_, thin-film solar cell, interface, transparent back contact, STEM, HAXPES

## Abstract

The effect of a thin sacrificial Mo interlayer on the device performance of a thin-film solar cell based on a wide band gap (1.46 eV) (Ag, Cu)­(In, Ga)­Se_2_ (ACIGSe)/indium tin oxide (ITO) layer stack and its influence on the chemical composition at the absorber/transparent back contact (TBC) interface are investigated. The solar cell without a Mo interlayer exhibits an efficiency of less than 1%, whereas the inclusion of an approximately 10 nm thick Mo interlayer produces a cell with an efficiency of 10.1%, with the fill factor being more than tripled. Scanning transmission electron microscopy and energy-dispersive X-ray spectroscopy line scans show strong GaO_*x*_ formation at the absorber/transparent conductive oxide (TCO) interface in a Mo-free sample and a significant decrease in the amount of GaO_*x*_ in a sample with the Mo interlayer included. Cleaving the absorber/TBC interface allowed us to study the absorber back side and the exposed ITO TCO using hard X-ray photoelectron spectroscopy. The nearly complete conversion of the metallic Mo interlayer into MoSe_2_ and MoO_*x*_ is revealed. It is suggested that the formation of MoO_*x*_, which may act as a hole-selective contact, together with inhibited GaO_*x*_ formation might be responsible for the pronounced performance enhancement for the cell with the thin Mo interlayer.

## Graphical abstract

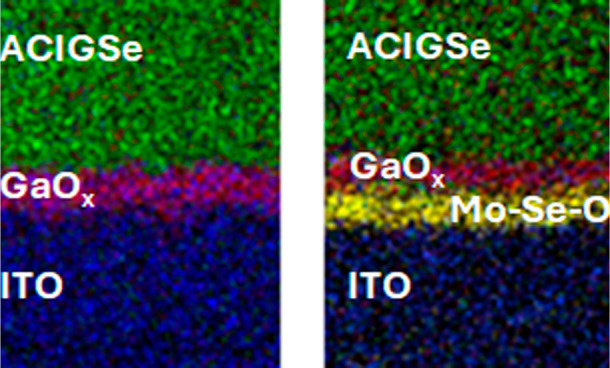

## Introduction

In Cu­(In, Ga)­Se_2_ (CIGSe)-based thin-film solar cells, opaque molybdenum (Mo) is commonly used as the back contact. At elevated temperatures upon absorber deposition under a selenium atmosphere, a MoSe_2_ interlayer is formed,^1^ which is responsible for the quasi-Ohmic contact, leading to large fill factors (FFs).^2^ However, for applications such as bifacial devices or top cells of multi-junction devices, transparent back contacts (TBCs) are required. For such purposes, various transparent conductive oxides (TCOs) have been investigated, including ZnO/Al (AZO), SnO/F (FTO), In_2_O_3_:Sn (ITO), and In_2_O_3_:H.^3–5^ While the deteriorated performance of devices based on CIGSe/FTO was attributed to the loss of fluorine at absorber deposition temperatures above 520 °C,^4^ all other TCO-based TBCs suffer from the formation of gallium oxide at the absorber/TCO interface at similar CIGSe deposition temperatures. Stoichiometric, crystalline β-Ga_2_O_3_ is an ultrawide band gap semiconductor (with a band gap energy, *E*_g_ = 4.9 eV).^6^ Its optoelectronic properties are expected to certainly affect the electronic structure at the TBC. In most cases, this gallium oxide at the interface is found to be substoichiometric (→ GaO_*x*_), and the precise effect of its formation at the TBC on the device performance is still under debate. The work of Heinemann et al. strongly suggests that acceptor as well as donor states may play a decisive role with respect to the electronic structure of GaO_*x*_,^3^ as recently also spectroscopically corroborated by Valenta et al.^7^ Oxygen-deficient and therefore n-doped GaO_*x*_ at the back contact could form a reverse junction at the back interface, affecting charge carrier transport.^4^ The formation of significant amounts of GaO_*x*_ at the TBC may also cause Ga depletion toward the back of the absorber, which negatively impacts the desired electric field, repelling electrons from the TBC.^5^ One opportunity to reduce or even control the formation of GaO_*x*_ at the absorber/TBC interface is the addition of a thin (a few tens of nm), metallic Mo interlayer.^4,8^ Reflectance measurements suggested that a 5 to 15 nm thick Mo interlayer sputter deposited on an ZnO/Al back contact is mainly converted to MoSe_2_ during CIGSe absorber deposition at elevated temperatures.^9^ Energy-dispersive X-ray spectroscopy (EDS) and scanning transmission electron spectroscopy (STEM) measurements showed similar results for an ITO back contact.^10^ Considering the (wide band gap) CIGSe absorber/TCO stack as part of a top cell in a tandem device, a pronounced MoSe_2_ interlayer with a smaller indirect (direct) band gap of about 1.1 eV^11–13^ (1.4 eV^11,14,15^ ) and/or a significant remainder of the metallic Mo may deteriorate the transparency of the TBC. In addition, neither study clearly addressed the question on how the Mo interlayer addition affects the GaO_*x*_ formation or could exclude the formation of additional chemical species such as MoO_*x*_.

In this study, we examine the effect of a thin sacrificial Mo interlayer on the device performance of a thin-film solar cell based on a wide-band-gap (Ag,Cu)­(In,Ga)­Se_2_ (ACIGSe)/ITO layer stack. While a cell without such an interlayer shows a poor power conversion efficiency (PCE) of less than 1%, a similar cell with an about 10 nm thick Mo layer deposited on the ITO prior to absorber deposition exhibits an efficiency of 10.1%, with the fill factor (FF) being more than tripled. A combination of STEM/EDS and HAXPES (hard X-ray photoelectron spectroscopy) measurements shows that GaO_*x*_ forms at the back contact of both samples; however, the layer thickness is significantly reduced upon Mo insertion. Analysis of the chemical composition of the interface suggests that metallic Mo is nearly completely converted to MoSe_2_ and (mainly) MoO_*x*_. Since the latter, much more dominant component is a prominent material to realize charge carrier selective contacts for holes,^16,17^ we suggest its formation together with inhibiting the formation of GaO_*x*_ as an explanation for the drastically enhanced cell performance. These findings provide crucial insights for the optimization of absorber/TCO interfaces in (A)­CIGSe-based bifacial or tandem devices.

## Experimental Section

### Sample Preparation and Handling

At ZSW, wide band gap ACIGSe absorbers were grown with an industry-relevant 30 × 30 cm^2^ in-line coater on TCO/glass substrates with substrate heater temperatures around 680 °C; therefore, we estimate the substrate temperature to be around the softening point of the glass substrates. Ag, Cu, In, Ga, and Se were coevaporated in a multistage process with a subsequent RbF postdeposition treatment (PDT) without breaking the vacuum. Further details on the ACIGSe deposition setup and process can be found in Gutzler et al.^18^ The polycrystalline ACIGSe absorbers are 2.4 μm thick with a GGI = [Ga]/([Ga] + [In]) of 0.73, an ACGI = ([Ag] + [Cu])/([Ga] + [In]) of 0.72, and an AAC = [Ag]/([Ag] + [Cu]) of 0.09, as measured by X-ray fluorescence. Two kinds of substrates were used as the back electrode: (1) a commercial 1 mm thick soda-lime glass (SLG) with ITO on the top (Solems S.A., SOL12) and (2) the same ITO/SLG substrate with an additional 10 ± 1 nm thin sputtered Mo layer on the top, as confirmed by profilometry measurements. Confocal microscopy images indicate the formation of a conformal Mo layer (on that scale) completely covering the relatively smooth ITO layer (Figure S1 in the Supporting Information and related discussion). After ACIGSe deposition, samples were annealed in air for 5 min at 200 °C and then coated with a ∼50 nm thick CdS buffer grown by chemical bath deposition. An rf-sputtered Zn_0.85_Mg_0.15_O high-resistive layer and a pulsed dc-sputtered AZO window layer make up the front electrode of the cell. The solar cells with a total area of 0.5 cm^2^ were completed with Ni/Al/Ni grid fingers without antireflective coating.

At the Iberian Nanotechnology Laboratory, cross-sectional samples were prepared using a FEI Helios NanoLab 450S focused ion beam (FIB) to have electron-transparent lamellae (thickness < 100 nm) for subsequent STEM/EDS analysis. The FIB operates with a gallium (Ga) liquid metal ion source, which is the reason why we deliberately abstained from quantifying the Ga content in the analyzed samples.

At HZB, the individual layer stacks were separated and cleaved at the ACIGSe/ITO or ACIGSe/thin Mo/ITO interfaces as described in ref 19. As a preliminary step, a layer of gold, some tens of nanometers thick, was deposited on the absorber surface via sputtering to act as a diffusion barrier against the silver from the conductive epoxy used to glue the samples onto a stainless-steel plate. After the epoxy/samples were cured for 24 h at 80 °C, the samples were immersed in liquid nitrogen. The resulting thermal stress in combination with applied mechanical stress resulted in cleaving the layer stack mainly along the ACIGSe/ITO or ACIGSe/thin Mo interface; the ACIGSe absorber film remained fixed to the stainless-steel plate via conductive epoxy. The cleaving is necessary to make the absorber/ITO interface, which is deeply buried below 2.4 μm ACIGSe, accessible for HAXPES measurements as their information depth is limited by the inelastic mean free path (IMFP) of the detected photoelectrons, which is at maximum approximately 3 nm when using an excitation energy of 2 keV.^20^ Both halves of each sample were mounted in ambient conditions (air exposure for approximately 15 min, with condensation of moisture potentially enhanced by the low sample temperature; surface moisture would largely be removed in the ultrahigh vacuum system but may affect the chemical surface structure) and transferred into the surface analysis system for HAXPES measurements.

### Characterization

Current density–voltage (*J*–*V*) characteristics were measured with an AAA WACOM solar simulator under standard testing conditions with a simulated AM1.5G spectrum. The light intensity was 100 mW/cm^2^, as calibrated with a silicon reference cell. The four-point probe geometry was used for contacting the ACIGSe-based devices, which were measured in the as-grown state without any additional light-soaking procedures.

External quantum efficiency (EQE) was measured with a Bentham PVE 300 setup. The EQE was used for extracting the optical band gap energy (*E*_g_) of the ACIGSe absorber (extrapolated from the (*E* × EQE)^2^ vs *E* plot, see Figure S2 in the Supporting Information).

Scanning transmission electron microscopy (STEM) coupled with energy-dispersive X-ray spectroscopy (EDS) experiments were performed to study the chemical composition of the FIB prepared cross-sections. High-angle annular dark field (HAADF) STEM images were acquired using a double-corrected FEI Titan G3 Cubed Themis 60–300 keV operated at 200 keV. The images of 2048 × 2048 pixels were recorded using a convergence angle of 21 mrad with a pixel dwell time set to 16 μs. EDS mapping was performed with the same instrument by using a Super-X EDS detector. Iterative maps of 2048 × 2048 pixels were recorded with a dwell time per pixel of 2 μs at 200 keV under a current between 150 and 250 pA and collection times of 15 min. The Velox software from Thermofisher Scientific was used for acquiring and processing the STEM–EDS data. The element-specific X-ray fluorescence lines used for analysis are listed in Table 1.

**Table 1:** Element-Specific X-ray Fluorescence Lines Used for EDS Analysis

element	line	energy of line (keV)
O	K	0.52
Cu	L	0.93
Ga	K	9.25
Se	K	11.22
Ag	L	2.98
In	L	3.29
Sn	L	3.44

Synchrotron-based hard X-ray photoelectron spectroscopy (HAXPES) experiments were conducted at the HiKE endstation located at the KMC-1 bending magnet beamline of the BESSY-II electron storage ring.^21^ This endstation was equipped with a Scienta R4000 electron energy analyzer with an entrance cone perpendicular to the incoming beam in the horizontal plane (i.e., polarization plane of the X-rays), and its base pressure was <1 × 10^–8^ mbar. The spectra were recorded in near-grazing incidence geometry with a photon energy of 2003 eV (for simplicity referred to in the manuscript as 2 keV) using the first-order diffraction of the Si(111) double-crystal monochromator. Small deviations from the nominal photon energy were corrected by referencing the Au 4f_7/2_ peak of a grounded clean Au foil to a binding energy of 84.00 eV.

Model Fit: The elemental surface composition was derived by evaluating the different peak intensities (i.e., peak areas) using the CasaXPS software.^22^ For all fits, we used pseudo-Voigt profile functions in combination with Shirley backgrounds. Spin–orbit doublets were fit with a pair of pseudo-Voigt functions with the same width and obeyed the 2*j* + 1 multiplicity rule. In spectra where multiple peaks overlapped, the energy separation between different spin contributions was fixed according to literature values.^23–26^ For quantification, the peak intensities were then corrected according to the IMFP of the photoelectrons, the photoionization cross-section,^27–29^ and the energy-specific transmission function of the analyzer.^30,31^

## Results and Discussion

Figure 1 depicts dark and light *J*–*V* curves of representative wide band gap ACIGSe solar cells (*E*_g_ = 1.46 eV) on ITO and thin Mo/ITO TBCs with the corresponding solar cell parameters listed in Table 2, and the respective EQE measured on a sister cell prepared on the same carrier is shown in Figure S2 in the Supporting Information. The nonfunctional cell on the ITO back contact with a power conversion efficiency (PCE) of 0.6% exhibits a strong blocking behavior (Figure 1a), whereas the cell based on the thin Mo/ITO TBC with a PCE of 10.1% shows the expected diode behavior (Figure 1b). The solar cell performance is mainly limited by a low FF of 62.2% (see Table 2), caused by a large series resistance of the thin Mo/ITO back contact and a minor shunt.

**Figure 1:**
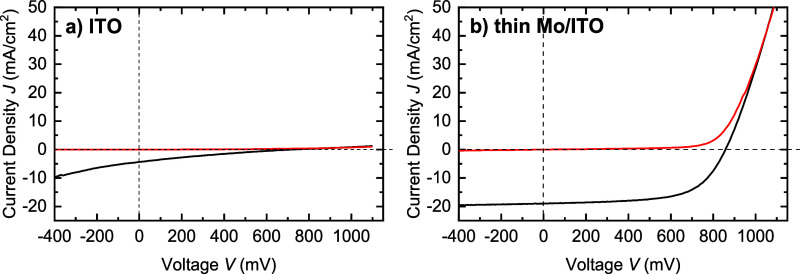
Dark (red line) and light (black line) *J*–*V* curves of wide band gap ACIGSe solar cells with absorber band gaps of 1.46 eV on different transparent back contacts. (a) ITO and (b) thin Mo/ITO.

**Table 2:** Corresponding Solar Cell Parameters (Power Conversion Efficiency [PCE], Open-Circuit Voltage [*V*_OC_], Fill Factor [FF], and Short-Circuit Current Density [*J*_SC_]) for Both Wide Band-Gap ACIGSe Cells Shown in Figure 1 with ITO and Thin Mo/ITO Back Contacts

	ACIGSe/ITO	ACIGSe/thin Mo/ITO
PCE [%]	0.6	10.1
*V*_OC_ [mV]	722	858
FF [%]	20.3	62.2
*J*_SC_ [mA/cm^2^]	4.4	19.0

The interface between the TBC and the ACIGSe absorber was characterized by STEM in combination with EDS. Figure 2 shows the HAADF-STEM image and EDS-derived chemical maps of both samples, ACIGSe/ITO and ACIGSe/thin Mo/ITO. The atomic fraction of prominent elements is shown in the EDS line profiles in Figure 2c for the ACIGSe/ITO and in Figure 2f for the ACIGSe/thin Mo/ITO stack. The ITO TBC can be clearly identified by the constant In signal and the similarly constant O signal between 0 and 120 nm. On the other side of the interface (i.e., between 160 and 250 nm), the ACIGSe absorber layer is clearly identified by the constant signals for Ga, In, and Se. For clarity, we excluded the profiles for Cu, Ag, and Sn (all profiles are shown in Figure S3 in the Supporting Information). The ∼150 eV energy resolution of EDS measurements causes an overlap of X-ray lines of different chemical elements in many cases, and thus, we do not attempt to quantify the chemical compositions based on the EDS line scans.

**Figure 2:**
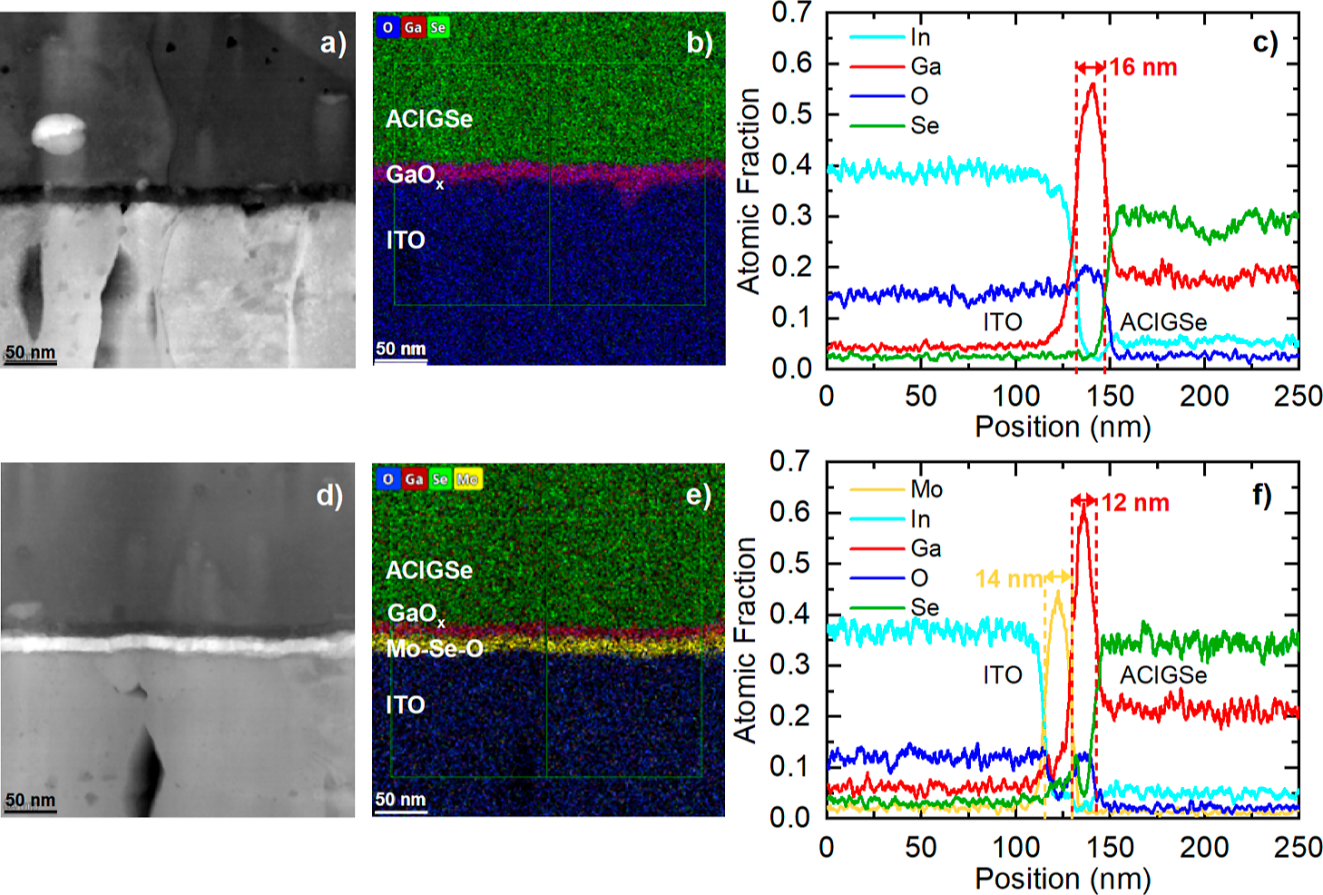
HAADF-STEM bright-field images (a,d), EDS-derived chemical maps (b,e), and EDS line profiles (c,f) of the ACIGSe-based solar cell layer stack with ITO (a, b, c) and thin Mo/ITO (d, e, f) back contacts, respectively.

Clear differences in the interface region (between about 120 and 160 nm) can be observed between the two samples, resulting from the presence of the ∼10 nm thick Mo interlayer on the ITO before the ACIGSe deposition process. For the Mo-free ACIGSe/ITO sample, a distinct increase in the Ga signal profile indicates a Ga-rich interlayer with a thickness of (16 ± 4) nm. This increase in the Ga signal coincides with an increase in the O signal, suggesting the formation of a GaO_*x*_ layer, in agreement with previous reports for CIGSe absorbers (without Ag) deposited on ITO TBC substrates.^4^

For the ACIGSe/thin Mo/ITO sample, a clear Ga peak coincident with an O peak indicates likewise the formation of a GaO_*x*_ layer; in this case, it is thinner: (12 ± 4) nm. This layer is adjacent to the ACIGSe absorber layer. Clearly separated between this GaO_*x*_ layer and the ITO TBC, a distinct peak in the Mo signal is observed, which suggests a (14 ± 4) nm thick Mo-rich layer slightly larger than the nominally deposited metallic Mo layer of (10 ± 1) nm. This Mo peak also overlaps with a decreased, but nonzero, O signal and an increased Se content (see Figure S4 in the Supporting Information). A Se signal variation is observed right at the interface between the Ga-rich and the Mo-rich interlayer, indicating the presence of MoSe_*x*_ and possibly also GaSe_*x*_ (where *x* indicates here likely deviations from the MoSe_2_ and Ga_2_Se_3_ stoichiometries and is not meant to specify a particular composition). The overlap of the Mo and O signals at the interface with ITO suggests the formation of MoO_*x*_ (see the statement on the meaning of *x* above).

It is noteworthy that for both samples, ACIGSe/ITO and ACIGSe/thin Mo/ITO, the observed interlayers between ITO and ACIGSe are spatially very well-defined, demonstrated by the consistent signals along the full length of the interface shown in the HAADF-STEM bright-field images (Figure 2a,d) and the EDS chemical maps (Figure 2b,e).

To complement the microscopy results with measurements having chemical speciation sensitivity, the TBC interfaces have also been studied by HAXPES. In order to make the deeply buried interfaces accessible to this more surface sensitive method, the ACIGSe/(thin Mo/)­ITO stacks were cleaved as described in the Experimental Section, and both sides (i.e., cleavage planes) were examined by HAXPES using an excitation energy of 2 keV. Figure 3 shows the HAXPES survey spectra of the ACIGSe absorber back sides and the exposed ITO. The absorber spectra contain peaks associated with the ACIGSe matrix elements Ag, Cu, In, Ga, Se, and Rb from the RbF-PDT. In addition, we find Na arising from the SLG substrate diffusing through the layer stack^32^ and peaks related to C and O, which we tentatively attribute to contamination taking place upon/after the cleaving procedure. There is little visible in these survey spectra that differentiates the two absorber backsides.

**Figure 3:**
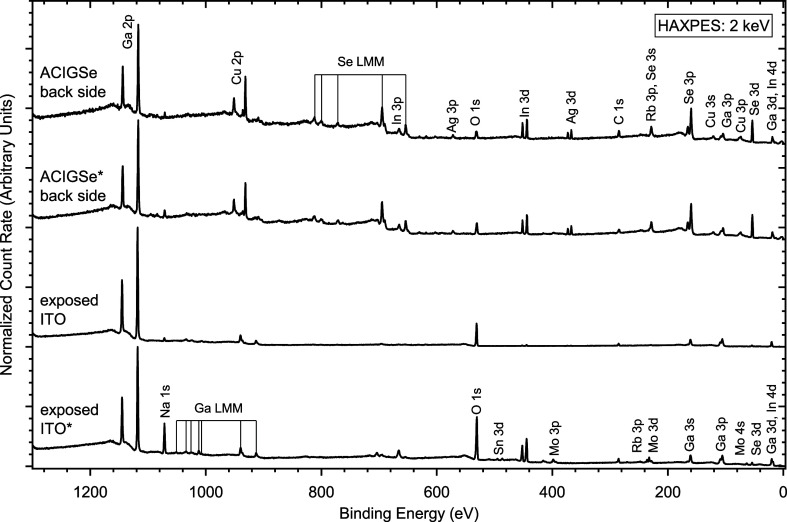
HAXPES survey spectra of the cleavage planes. The spectra marked with an asterisk are originally based on the TBC stack with the thin Mo interlayer. All spectra are normalized to the maximum intensity of the respective Ga 2p_3/2_ line. A vertical offset is added for the sake of clarity.

The two survey spectra of the exposed ITO differ in significant ways, indicating the strong influence of the thin Mo interlayer. The spectrum of the Mo-containing sample (indicated by * in Figure 3) is dominated by photoemission lines of the expected elements of ITO, namely, In, Sn, and O, along with significant contributions from Na and Ga. The peaks related to Mo are not prominent in the survey spectrum, mainly due to the small amount, the comparably low photoionization cross-section at this excitation energy,^27^ and the cleavage plane that leaves an attenuating GaO_*x*_ on the top of the Mo-layer. In the survey spectrum of the exposed ITO of the Mo-free sample, the In and Sn signatures are significantly decreased (almost disappear), and the spectrum is dominated by Ga- and O-related signals. Together with the STEM results, the survey spectra suggest that the main cleavage plane is between the absorber and the Mo for the ACIGSe/thin Mo/ITO sample (in agreement with earlier reports on the cleavage of absorber/Mo/glass samples^1,19,33^ ) and between the absorber and/or within the gallium-rich layer formed between the absorber and ITO (see Figure 2, lower panel) for the ACIGSe/ITO sample. However, the Mo 3d detailed spectrum of the ACIGSe back side (displayed in Figure S5 in the Supporting Information) indicates the presence of small amounts of Mo also on the absorber side. This could be either a result of Mo diffusion into the absorber or of non-uniform cleavage. Different cleavage scenarios have been discussed in the past.^1^

To determine the chemical environment of gallium in the GaO_*x*_ layer, we turn to the Ga 2p_3/2_ core level spectra measured on the cleavage planes of each layer stack and the respective peak fits shown in Figure 4. On both absorber back sides, the spectrum can be fitted with two species, a primary one at (1117.65 ± 0.05) eV and a secondary feature at (1118.73 ± 0.09) eV binding energy (BE). We assign the primary peak at lower BE to Ga–Se bonds of ACIGSe and the secondary peak at higher BE to Ga–O bonds.^34–36^ Note that the Ga–Se bond environment of the absorber back side of the initial TBC stack with the intermediate thin Mo layer would also be in line with the presence of a GaSe_*x*_ layer, as suggested by the EDS line profiles in Figures 2f and S3b and Figure S4 in the Supporting Information. The analysis of the corresponding modified Auger parameters of Ga (computed as the sum of the BE of the respective contribution to the Ga 2p_3/2_ peak and the kinetic energy, of the corresponding Ga L_3_M_45_M_45_ feature, depicted in Figure S6) presented in a Wagner plot in Figure S7 corroborate that the two Ga 2p_3/2_ components refer to two different chemical bonding environments: Ga–Se and Ga–O. Note that for the ACIGSe back side of the Mo-containing sample, the total intensity of the Ga 2p_3/2_ peak as well as the intensity ratio of the different contributions to the Ga 2p_3/2_ line (Ga–O/Ga–Se) is roughly double that of the absorber back side of the Mo-free sample.

**Figure 4:**
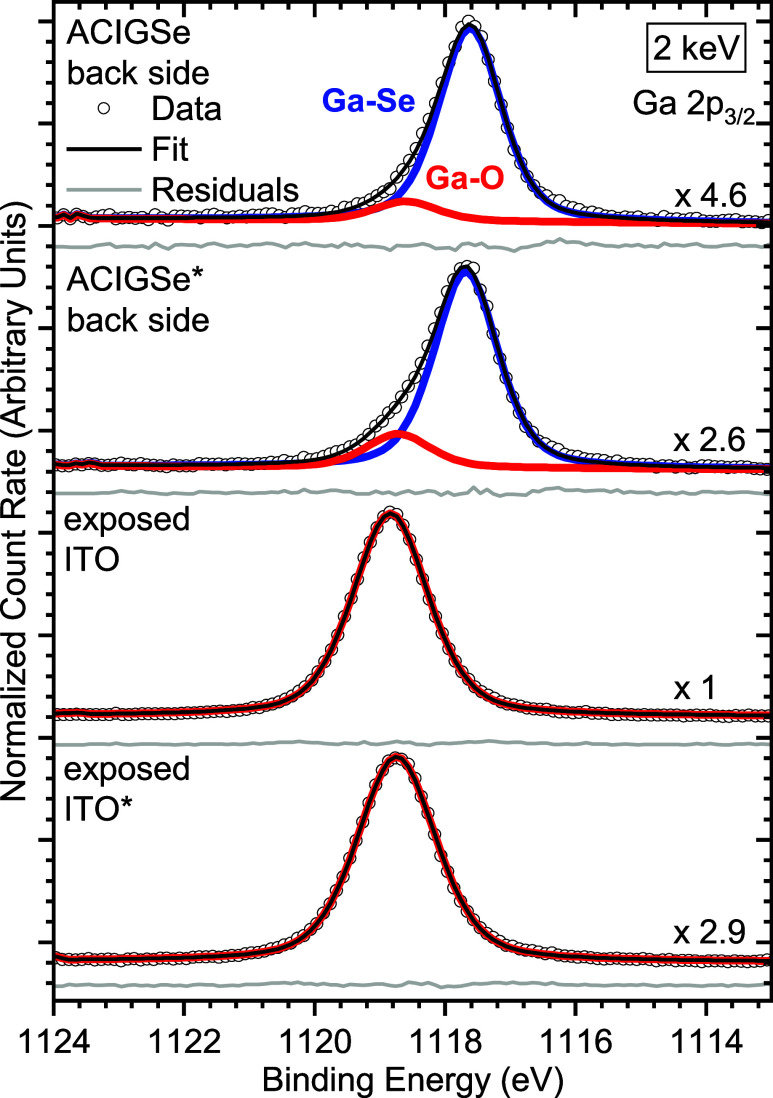
HAXPES detailed spectra of the Ga 2p_3/2_ region of the cleavage planes, including fits and the respective residuals. The * denotes the spectra of the samples of the TBC stack with the thin Mo interlayer. Vertical offsets are added for clarity. Note the different magnification factors.

In contrast to the Ga 2p_3/2_ spectra of the absorber backsides, on the exposed ITO side, only the higher BE Ga–O contribution to the Ga 2p_3/2_ is observed. Its intensity in the exposed ITO spectrum of the Mo-free sample is almost three times—note the different magnification factors in Figure 4—that of the corresponding peak in the Mo-containing sample, implying that while we find more Ga (and Ga–O) on the ACIGSe back side of the Mo-containing sample, on the corresponding exposed ITO, significantly less Ga–O is observed, meaning that the total amount of GaO_*x*_ is decreased.

These findings suggest that a gallium oxide layer is formed between the ITO and the absorber, and the presence of the thin Mo interlayer reduces its thickness—in agreement with the STEM analysis in Figure 2. This interpretation is also supported by the strong attenuation of the indium oxide related In 3d feature (red) in the core level spectra of the exposed ITO of the sample without a thin Mo interlayer presented in Figure S8 in the Supporting Information. While such features are clearly visible on the thin Mo/ITO sample, the gallium oxide layer on the bare ITO is apparently thick enough to reduce the photoemission signal from the indium oxide in the ITO by a factor of 6.7.

The HAXPES and STEM results strongly suggest that the gallium-rich layer at the absorber/TCO interface is predominantly gallium oxide, a finding that is in agreement with several previous studies.^3–5,9,10,37^ However, for the TBC stack with the thin Mo interlayer, the formation of GaSe_*x*_ can also not be excluded. In order to identify the stoichiometry of the gallium oxide, we determine the O/Ga ratio on the exposed ITO of the Mo-free layer stack based on the peak area below the respective Ga 2p_3/2_ (see Figure 4) and the O 1s peak shown in Figure S10. In order to avoid overestimating the O content of GaO_*x*_, we subtract the contribution of the ITO to the O 1s peak before calculating the O/Ga ratio. A detailed description of the calculation is given in the Supporting Information. Regarding the O 1s peak, note that more than 7% of the peak area is assigned to the O–H groups (see Figure S10), which likely results from a partial conversion of GaO_*x*_ into Ga­(OH)_3_ upon air and thus moisture exposure after the cleaving during sample mounting. Assuming that all the O–H groups are attributed to Ga­(OH)_3_, we derive an O/Ga ratio of 1.0 ± 0.2. This suggests that compared to the most common form of gallium oxide (Ga_2_O_3_) a significantly oxygen-deficient GaO_*x*_ forms at the absorber/TCO interface. The found O/Ga ratio implies a composition similar to the GaO_*x*_ (O/Ga = 1.1 ± 0.1) formed by oxidation of nanostructured metallic Ga in partial pressures of oxygen^36^ or GaO_*x*_ films sputter-deposited onto CIGSe absorbers in an argon atmosphere without additional oxygen.^7^ In the latter study, the authors show an almost flat conduction band alignment at the GaO_*x*_/CIGSe absorber interface, while the valence band offset (VBO) is strongly negative ([−3.21 ± 0.19] eV). While a somewhat less negative VBO can be expected for the (wide band gap) absorber/TCO back contact studied, it can also be expected that the VBO will still be significantly negative and thus detrimental for a transparent hole-selective contact. This energy level alignment situation, unsuited for achieving high PCE, might be mitigated by the (STEM and HAXPES indicated) presence of GaSe_*x*_, which has a lower band gap than GaO_*x*_. Moreover, Valenta et al. found defect states above the GaO_*x*_ valence band maximum, attributed to oxygen vacancies extending almost to the Fermi level.^7^ It is not clear at this point whether in the studied case, they are beneficial or detrimental to the device performance as they can act as recombination centers^38^ or allow for efficient transport of the photogenerated holes through the GaO_*x*_.

To reveal the chemical structure of thin Mo in the final cell stack, we discuss the Mo 3d core level region next. Figure 5 shows the detailed spectrum of the exposed ITO of the Mo-containing sample along with fits for different Mo 3d species and the Se 3s and Rb 3p_3/2_ core levels, all appearing in the same energy range. Three pairs of Mo 3d peaks are required to fit the spectrum reasonably well. They can be assigned to three different species of molybdenum. The Mo 3d_5/2_–3d_3/2_ doublet at (227.91 ± 0.05) and (231.07 ± 0.05) eV agrees with literature values for metallic molybdenum,^39^ the one at (228.85 ± 0.06) and (231.99 ± 0.06) eV with values found for MoSe_2_,^1^ and the peaks at (233.0 ± 0.2) and (236.1 ± 0.2) eV, respectively, can likely be attributed to Mo–O bonds with Mo being in an oxidation state of Mo^6+^, suggesting MoO_3_ as the predominant species.^40,41^ A determination of the O/Mo ratio was not possible since the sample also contains indium, tin, and gallium oxides whose contributions to the O 1s peak cannot be differentiated from that of MoO_*x*_. Assuming a homogeneous distribution of the different chemical species in the Mo layer, their relative intensities imply that the Mo is predominantly oxidized (78%); some Mo is selenized (18%); and only a small fraction is still metallic (4%). A similar XPS study conducted on a standard opaque Mo/glass back contact after lifting off the CIGSe absorber showed that only MoSe_2_ was formed.^1^ Thus, we propose that the main mechanism of MoO_*x*_ formation is related to the presence of ITO, which acts as an oxygen source, implying not only that the sacrificial interlayer is a physical barrier against GaO_*x*_ formation but also that MoO_*x*_ formation offers an alternative scavenger reaction path for oxygen from the ITO. The MoO_*x*_ should therefore reside predominantly next to the ITO, while the MoSe_2_ likely forms next to the absorber. This configuration could also explain the complicated EDS line profile in the interface region in Figure 2f. If this is the case, then MoSe_2_ would contribute more strongly, relative to its abundance, to our measured signal than MoO_*x*_, assuming that the cleavage left MoSe_2_ at the surface. The coexistence of MoSe_2_ and MoO_*x*_ might be beneficial for the intended application of the ACIGSe/ITO device as the top cell in a tandem configuration. While it has been suggested in the past that MoSe_2_ that forms upon absorber preparation between the chalcopyrite absorber and opaque Mo is responsible for the quasi-Ohmic back contact,^9,42–44^ the predominant conversion of the metallic Mo interlayer in direct contact with the ITO back contact into MoO_*x*_ (that has a wider band gap than MoSe_2_ ^45–48^ ) in our case will have a beneficial impact on the optical transparency of the ACIGSe/thin Mo/ITO stack. This relation enables the addition of a sacrificial interlayer of Mo as a viable approach to optimize the absorber/ITO back contact stack for tandem applications, even more so as MoO_*x*_ is a prominent material to realize charge carrier selective contacts for holes.^16,17,49^

**Figure 5:**
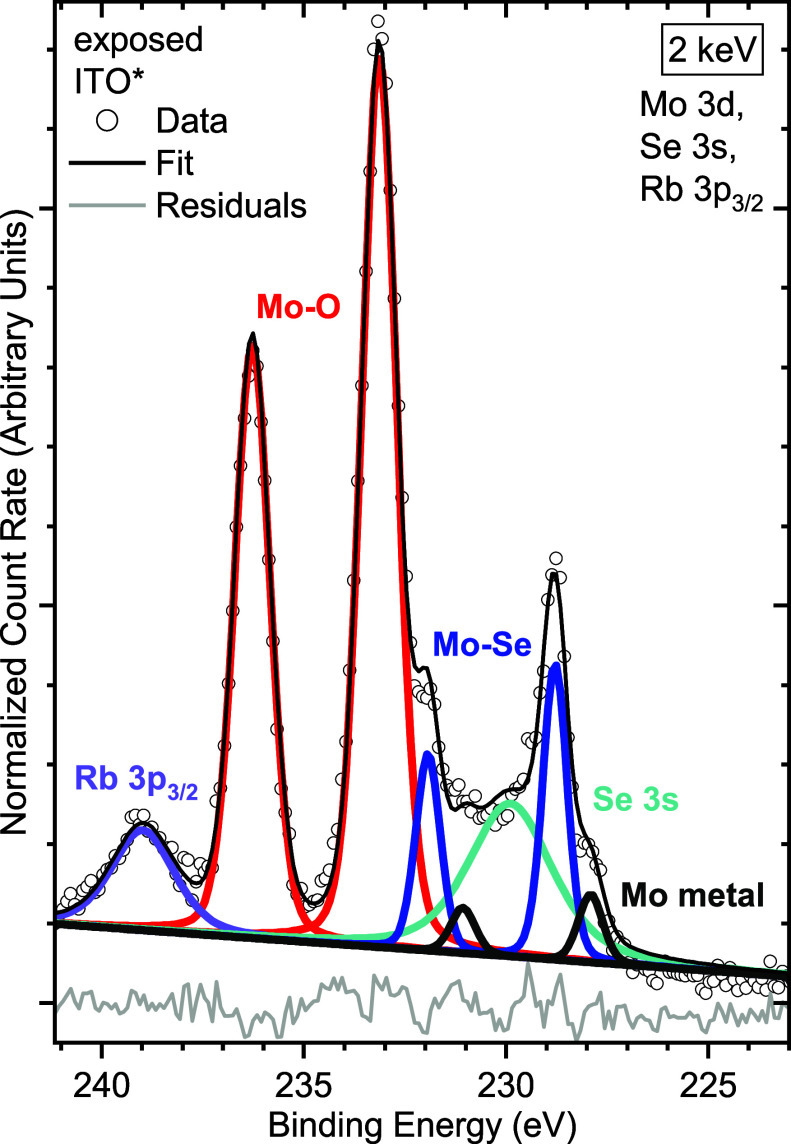
HAXPES detailed spectra of the Mo 3d region of the exposed ITO of the sample containing a thin Mo interlayer, including fits and respective residual. Note that the Mo 3d (partially) overlaps with the Rb 3p_3/2_ and Se 3s lines.

## Conclusions

This study investigates the effect of a thin sacrificial metallic Mo interlayer on the performance of a thin-film solar cell based on a wide band gap ACIGSe absorber/ITO layer stack and the chemical structure of the absorber/TBC interface. We find an enhancement of the PCE from less than 1% to >10% upon employing an about 10 nm thick Mo layer on the top of the ITO, followed by an ACIGSe absorber prepared by an industry-relevant process, with the FF being more than tripled. We attribute this large improvement in overall performance to a significant reduction in GaO_*x*_ formation at the absorber/TCO interface and almost complete conversion of the metallic Mo interlayer into MoSe_2_ but mainly MoO_*x*_. The formation of the latter offers an alternative reaction path to GaOx formation and seems especially beneficial for the wide band gap ACIGSe/ITO interface potentially used in a top cell of a tandem device as compared to MoSe_2_, it has a much larger band gap than ACIGSe (reducing parasitic absorption losses) and is a prominent material for realizing hole-selective contacts. This finding suggests that exploiting the concept of a sacrificial metallic Mo interlayer at the absorber/TCO interface could be a viable route for the further improvement of cell performance.

## Supplementary Material



## Data Availability

The raw data has been deposited online in a Zenodo repository and has the following permanent DOI: https://doi.org/10.5281/zenodo.14587753.

## Notes

Confocal micrographs of the pristine ITO sample and ITO with an about 10 nm thick Mo layer on top, EQE data measured on a sister cell with thin Mo/ITO TBC prepared on the same carrier during ACIGSe absorber deposition, STEM-EDS line profiles shown in Figure 2, f including Cu, Sn, and Ag STEM-EDS line profiles of the ACIGSe/thin Mo/ITO layer stack shown in Figure 2f zoomed in into the 95–160 nm region, description of thickness quantification in STEM, additional HAXPES spectra, Wagner plot for gallium, and description of elemental ratio calculations from HAXPES data (PDF)
