# Supplementary material for: Impact of a Thin Sacrificial Mo Layer on the Formation of the Wide Band Gap ACIGSe Absorber/ITO Thin-Film Solar Cell Interface
Source: ACS Appl Mater Interfaces. 2025 May 20;17(22):33027–35. doi: 10.1021/acsami.5c02315 (PMC12147069; doi:10.1021/acsami.5c02315)
Supplement: Supplementary file 1 [file am5c02315_si_001.pdf]

Supporting information

Impact of a thin sacrificial Mo layer on the formation of the wide band-gap ACIGSe absorber/ITO thin-film solar cell interface

Angelika Demling[1], Rico Gutzler[2], Cristiana Filipa Almeida Alves[3], Regan G. Wilks[1,4], Roberto Félix[1], Dimitrios Hariskos[2], Stefan Paetel[2], Rafael Cerqueira[3], Sascha Sadewasser[3], Wolfram Witte[2], Marcus Bär[1,4,5,6*]*

[1]Department of Interface Design, Helmholtz-Zentrum Berlin für Materialien und Energie GmbH (HZB), 12489 Berlin, Germany, email: angelika.demling@helmholtz-berlin.de; marcus.baer@helmholtz-berlin.de

[2]Zentrum für Sonnenenergie- und Wasserstoff-Forschung Baden-Württemberg (ZSW), 70563 Stuttgart, Germany

[3]International Iberian Nanotechnology Laboratory (INL), 4715-330 Braga, Portugal

[4]Energy Materials In-Situ Laboratory Berlin (EMIL), HZB, 12489 Berlin, Germany

[5]Department of X-ray Spectroscopy at Interfaces of Thin Films, Helmholtz-Institute Erlangen-Nürnberg for Renewable Energy (HI ERN), 12489 Berlin, Germany

[6]Department of Chemistry and Pharmacy, Friedrich-Alexander-Universität Erlangen-Nürnberg (FAU), 91058 Erlangen, Germany

Surfaces of pristine ITO and ITO with a thin Mo layer were optically studied using a Keyence VK-9700 3D Laserscan Microscope. Areas with high 'defect' density were chosen as to allow for some visual contrast in the images. The high resolution in the vertical direction of the order of 1 nm should allow to distinguish between conformal and island growth of the sputter deposited Mo layer. For reference, Figure S1a) shows the smooth surface of the pristine ITO without Mo layer. Figure S1b) shows the likewise smooth Mo layer on ITO. Neither a high roughness of the surface, nor differences in contrast between Mo-coated and uncoated parts of the surface can be detected, indicating a conformal coverage.

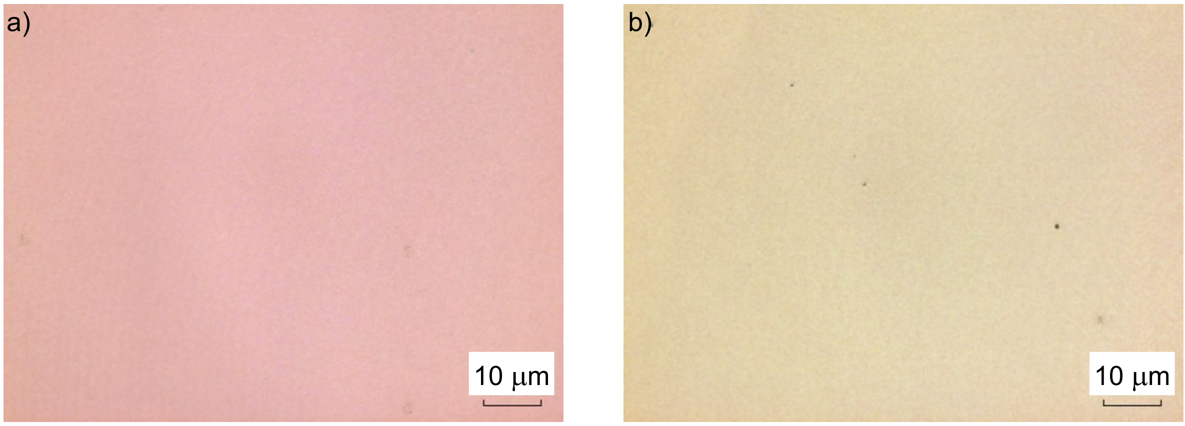

Figure S 1: Confocal micrographs of a) pristine ITO sample and b) ITO with an about 10 nm thick Mo layer on top.

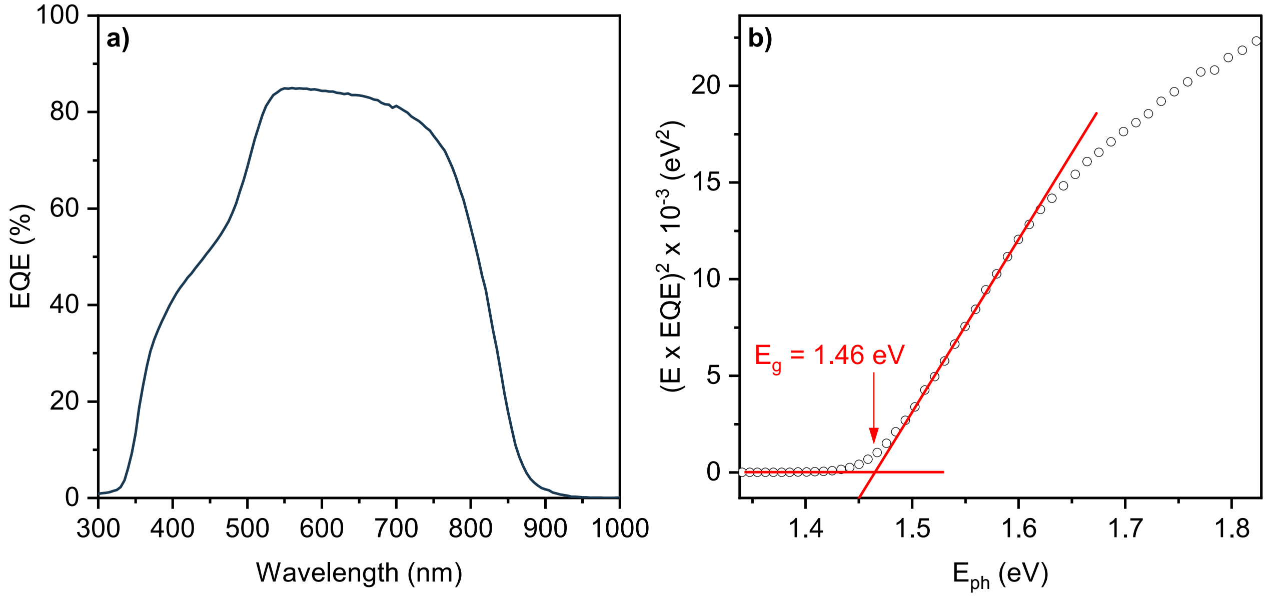

Figure S2: a) EQE data measured on a wide band-gap sister cell with the thin Mo/ITO TBC prepared on the same carrier during ACIGSe deposition as the samples studied by STEM and HAXPES and discussed in the manuscript. b) Plot of $(E \times EQE)^2$ versus photon energy (E_{ph}). The red lines indicate the extrapolation of the onset to 0 which is used to determine the band-gap energy of the ACIGSe absorber (E_g) to be 1.46 eV.

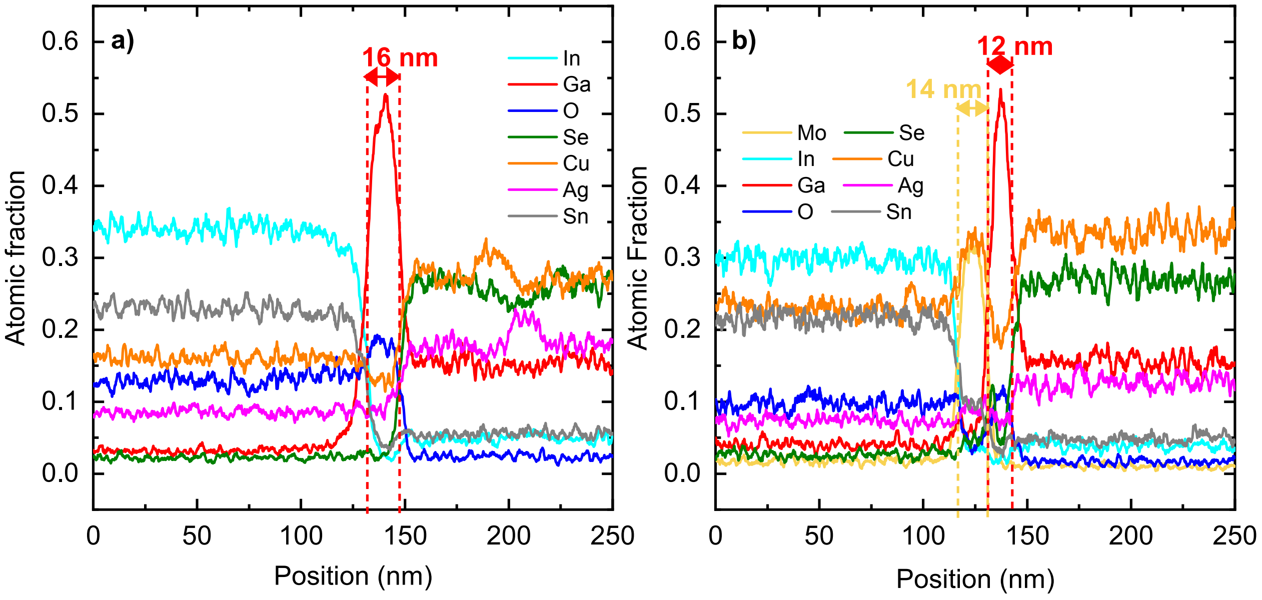

Figure S3: STEM-EDS line profiles (incl. Cu, Ag, and Sn) of the a) ACIGSe/ITO and b) ACIGSe/thin Mo/ITO layer stacks. Line profiles were extracted from the EDS maps shown in Figure 2 in the main manuscript.

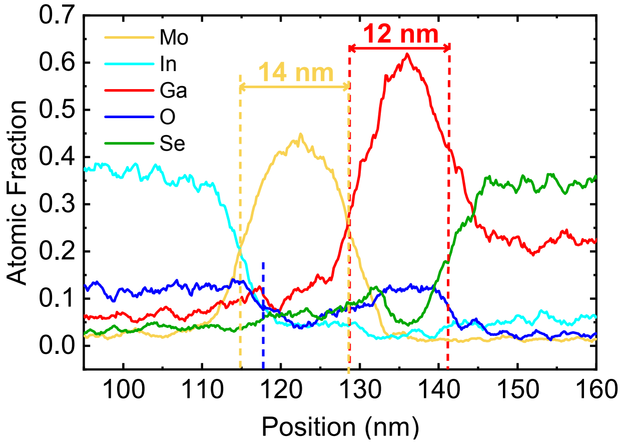

Figure S4: STEM-EDS line profile of the ACIGSe/thin Mo/ITO layer stack (shown in Figure S3b) zoomed in into the 95 – 160 nm region.

The thickness of the interface layers was determined by the "full width at half maximum (FWHM)", considering the half maximum on each side of the peak, as the Ga atomic fraction is different between the two sides of the interface. To determine the uncertainty in the thickness value, we determined the full width at 75% and 25% of the maximum.

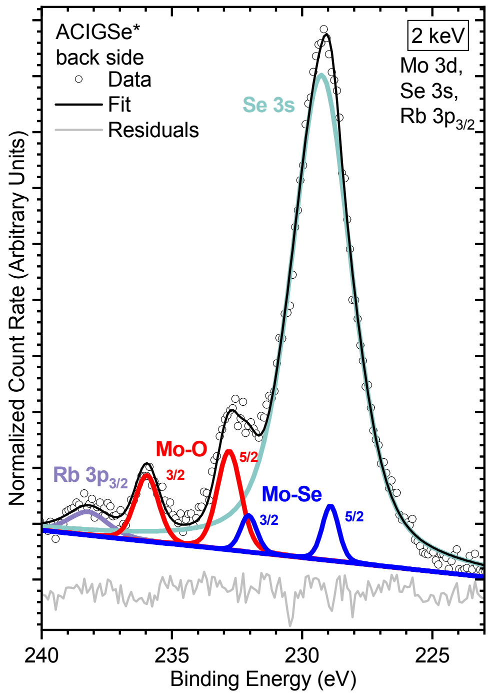

Figure S5: The HAXPES detail spectra of the Mo 3d region of the wide band-gap ACIGSe absorber back side of the sample containing a thin Mo interlayer, including fits, and respective residual. Note that the Mo 3d partially overlaps with the Rb $3p_{3/2}$ and Se 3s lines.

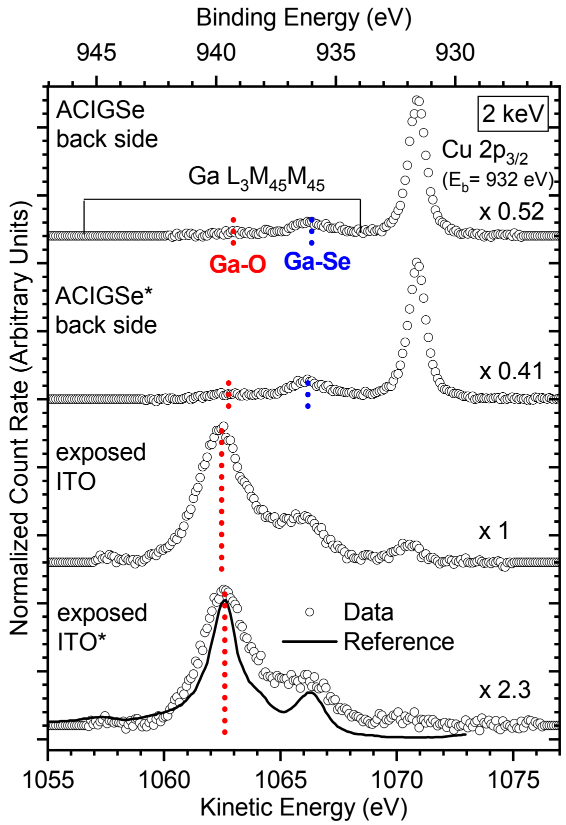

Figure S6: The HAXPES detail spectra of the Ga $L_3M_{45}M_{45}$ Auger peak region (overlapping with the Cu $2p_{3/2}$ line) of the cleavage planes, compared to the spectrum of elemental gallium from reference[1] shifted according to the main peak in the spectrum of the Mo-containing exposed ITO to achieve best overlap. Vertical offsets are added for clarity. The dashed lines mark the centers of the Ga $L_3M_{45}M_{45}$ peaks for the Ga-Se (blue) and Ga-O (red) species. The samples marked with * are originally based on the TBC stacks with the thin Mo interlayer. Note the different magnification factors.

Figure S6 displays HAXPES detail spectra of the Ga $L_3M_{45}M_{45}$ region near the Cu $2p_{3/2}$ peak. For both absorber backsides (two top spectra), the spectra are dominated by the pronounced Cu feature. Toward lower kinetic energy, two weaker features appear which we assign to the main Ga

$L_3M_{45}M_{45}$ peak arising from two different chemical species labelled with Ga-Se and Ga-O in agreement with the Ga $2p_{3/2}$ peaks shown in Figure 4 in the main text. In contrast to that in the spectra measured on both exposed ITO TBCs, the Cu $2p_{3/2}$ peak is very weak, likely arising from residual absorber material. Instead, the Ga $L_3M_{45}M_{45}$ is much more pronounced. Taking the spectral shape of the Ga $L_3M_{45}M_{45}$ feature (see direct comparison with a digitized reference spectrum measured on elemental Ga[1], black line) into account, we assign both Ga $L_3M_{45}M_{45}$ spectra to Ga-O bonds in agreement with our interpretation of the Ga $2p_{3/2}$ spectra.

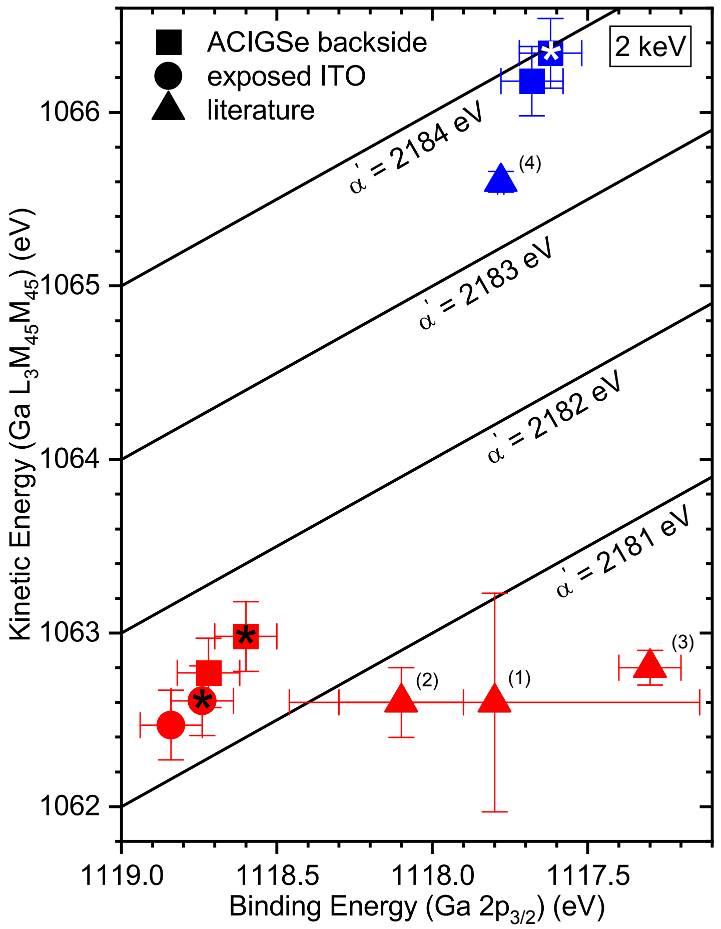

Figure S7: Wagner plot of the modified Auger parameter α′ (Ga) = $BE^{Ga\ 2p3/2}$+$KE^{Ga\ L3M45M45}$, as derived for the studied cleavage planes and literature values for (1) & (2) Ga_2O_3[2,3], (3) GaO_x[4], and (4) CIGSe[5]. The symbols marked with * represent data from samples originally based on the TBC stacks with the thin Mo interlayer. The blue symbols indicate the modified Auger parameters for the Ga $2p_{3/2}$ and Ga LMM contribution ascribed to Ga-Se and the red symbols to those of Ga-O. The error margin of our experimental data is ± 0.1 eV in x and ± 0.2 eV in y-direction.

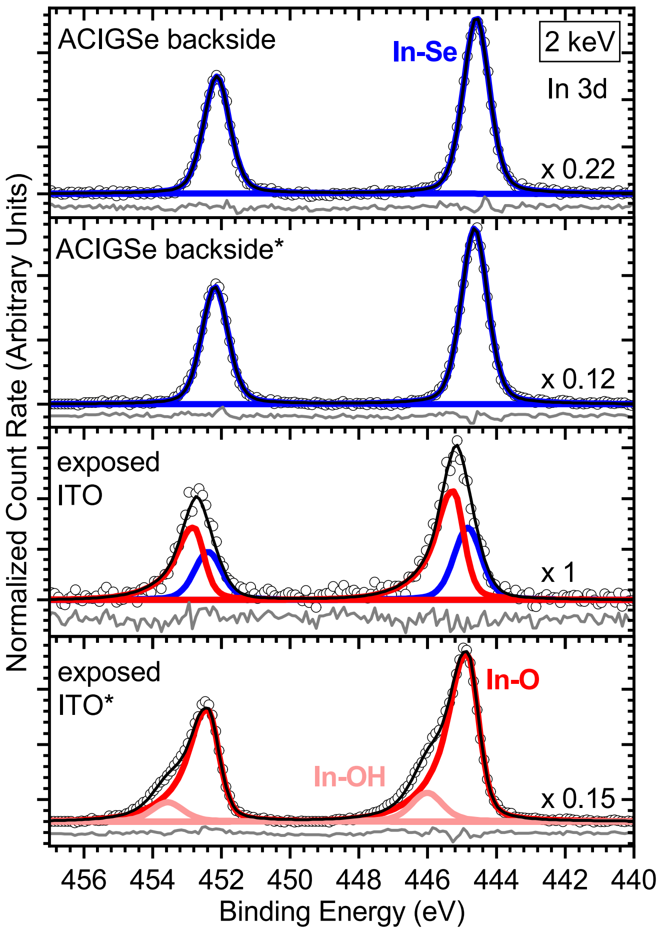

Figure S8: The HAXPES detail spectra of the In 3d region of the exposed ITO after cleavage, including fits, and respective residuals. Vertical offsets are added for clarity. The samples marked with * are originally based on the TBC stacks with the thin Mo interlayer. Note the different magnification factors.

For both exposed ITO sides, independent of the addition of a thin Mo interlayer, we find an asymmetric contribution to the In 3d line which we attribute to In-O bonds characteristic for degenerately doped (i.e., metal-like) ITO. A direct comparison of the In 3d signal intensity reveals an attenuation of the signal by 6.7 for the ITO of the Mo-free layer stack. The In 3d fit of the ITO

side of the layer stack with a Mo interlayer indicates the presence of In-OH bonds, probably caused by the short exposure to ambient conditions during sample mounting. On the ITO of the Mo-free layer stack, this signal is not as prominent. Presumably, the formation of In-OH bonds during short air exposure is suppressed by the formed thick gallium oxide layer. Instead, we find an additional low binding energy In 3d component, which we ascribe to the presence of In-Se bonds mainly caused by residual absorber material. However, the In/Cu (Se/Cu) ratio of 0.8 ± 0.1 (13 ± 2) derived from the In $3d_{5/2}$ (Se $3d_{5/2}$) and Cu $2p_{3/2}$ spectra of the exposed ITO is significantly higher than the one determined for the respective absorber backside 0.52 ± 0.05 (4.0 ± 0.6), which is close to the nominal [bulk] ratios of 0.38 (3.1), suggests an additional (partial) selenization of the ITO during absorber formation for the sample without Mo interlayer. This is also corroborated by the Se 3d XPS spectra discussed below. Note that the presence of a small In-Se contribution to the In 3d fit of the exposed ITO of the layer stack with a Mo interlayer indicates that the presence of a small quantity of residual absorber material cannot be excluded.

The Se 3d doublet on both absorber back sides displayed in the upper half of Figure S9 indicates the presence of one (main) Se species and agrees well with results on similar, but Ag-free absorber backside samples in reference[6] and is assigned to selenium in Cu chalcopyrite. Surprisingly, small Se 3d signals (note the scaling factors) are also detected on the exposed ITO surfaces shown in the lower half of Figure S9, corroborating the presence of residual absorber material on both exposed ITO sides. The spectral shape of the Se 3d of the exposed ITO of the layer stack with a Mo interlayer exhibiting significant spectral intensity at high BE (> 55.5 eV) might be attributed to the presence of $Se\text{-}O_x$ bonds probably formed during air exposure after sample cleaving. The more

complex spectral shape of the Se 3d of the ITO of the Mo-free layer stack indicates the presence of an additional Se species in agreement with the (partial) selenization of the ITO discussed for this sample above. Note that we deliberately abstain from fitting the Se 3d because of this complex chemical structure and the absence of an appropriate model.

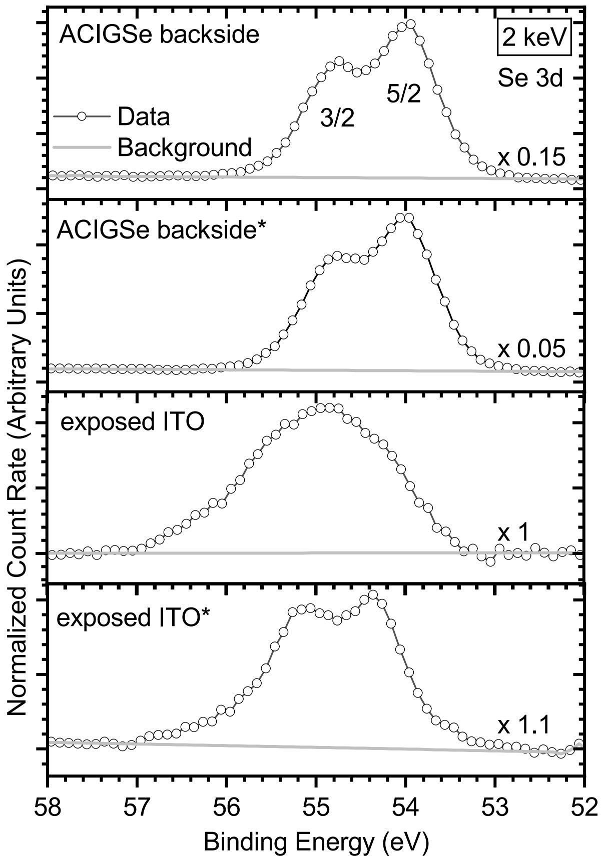

Figure S9: The HAXPES detail spectra of the Se 3d detail spectra of the different samples, including fits, and respective residual. Vertical offsets are added for clarity. The samples marked with * are originally based on the TBC stacks with the thin Mo interlayer. Note the different magnification factors.

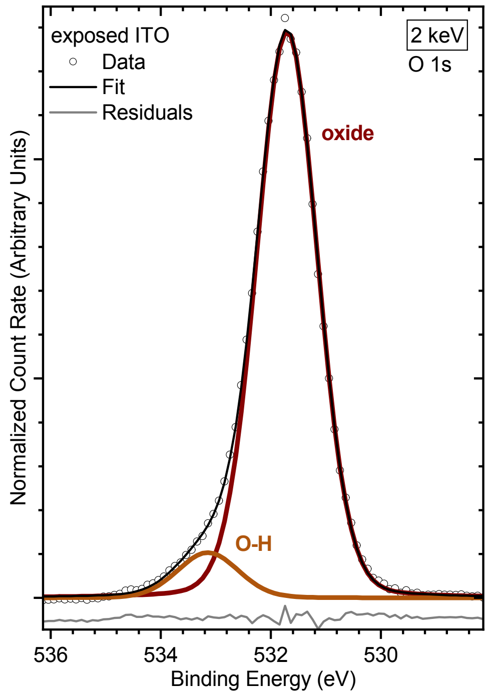

Figure S10: The HAXPES detail spectra of O 1s region of the exposed ITO of the Mo-free sample, including fits, and respective residual.

To calculate the elemental ratios from the measured spectra, the measured intensities were compared to those of a "virtual reference sample" calculated using SESSA version 2.2. The SESSA simulation takes as input the sample stoichiometry, experimental geometry, and excitation energy, and calculates peak intensities accounting for photoionization cross section, inelastic mean free path (IMFP), and other physical effects influencing the photoemission intensity[7–9]. The kinetic energy dependent transmission function of the photoelectron analyzer was calculated according to

reference [10,11], as it is not accounted for in SESSA. The respective values for each peak are shown in Table S1. The measured peak intensities determined from the respective fits were divided by the simulated intensities as well as the respective values of the analyzer transmission function before the elemental ratios were calculated.

For the determination of the O/Ga ratio of the GaO_x layer on the ITO of the Mo-free sample, a fraction of the O 1s signal is subtracted to account for the presence of In-O bonds arising from the ITO. This fraction is calculated based on a nominal In_2O_3/SnO_2 weight ratio of 9:1.

Table S1: Quantification parameters for the evaluation of the listed core levels.

	Ga $2p_{3/2}$	O 1s	Se $3d_{5/2}$	Cu $2p_{3/2}$	In $3d_{5/2}$
Simulated peak intensity for a sample with Ga/O/Se/Cu/In = 1	2.3e-05	4.8e-06	2.0e-06	2.0e-05	1.9e-05
Analyzer transmission function	0.84	0.75	0.68	0.81	0.73

REFERENCES

(1) Moulder, J. F.; Stickle, W. F.; Sobol, P. E.; Bomben, K. D. Handbook of XPS.Pdf. 1995, p 255.

(2) Bourque, J. L.; Biesinger, M. C.; Baines, K. M. Chemical State Determination of Molecular Gallium Compounds Using XPS. *Dalt. Trans.* **2016**, *45* (18), 7678–7696. https://doi.org/10.1039/c6dt00771f.

(3) Schön, G. Auger and Direct Electron Spectra in X-Ray Photoelectron Studies of Zinc, Zinc Oxide, Gallium and Gallium Oxide. *J. Electron Spectros. Relat. Phenomena* **1973**, *2* (1), 75–86. https://doi.org/10.1016/0368-2048(73)80049-0.

(4) Wagner, C. D. Chemical Shifts of Auger Lines, and the Auger Parameter. *Faraday Discuss. Chem. Soc.* **1975**, *60*, 291–300. https://doi.org/10.1039/dc9756000291.

(5) Majumdar, I.; Parvan, V.; Greiner, D.; Schlatmann, R.; Lauermann, I. Effect of Na from Soda-Lime Glass Substrate and as Post-Deposition on Cu(In,Ga)Se_2 Absorbers: A Photoelectron Spectroscopy Study in Ultra-High Vacuum. *Appl. Surf. Sci.* **2020**, *514*, 145941. https://doi.org/10.1016/j.apsusc.2020.145941.

(6) Bombsch, J.; Avancini, E.; Carron, R.; Handick, E.; Garcia-Diez, R.; Hartmann, C.; Félix, R.; Abou-Ras, D.; Ueda, S.; Wilks, R. G.; Bär, M. Unraveling the Impact of Combined NaF/RbF Postdeposition Treatments on the Deeply Buried Cu(In,Ga)Se_2/Mo Thin-Film Solar Cell Interface. *Adv. Energy Sustain. Res.* **2021**, *2* (11), 2100101. https://doi.org/10.1002/aesr.202100101.

(7) Trzhaskovskaya, M. B.; Nefedov, V. I.; Yarzhemsky, V. G. Photoelectron Angular Distribution Parameters for Elements Z=1 to Z=54 in the Photoelectron Energy Range 100–5000 EV. *At. Data Nucl. Data Tables* **2001**, *77* (1), 97–159. https://doi.org/10.1006/adnd.2000.0849.

(8) Trzhaskovskaya, M. B.; Nikulin, V. K.; Nefedov, V. I.; Yarzhemsky, V. G. Non-Dipole

Second Order Parameters of the Photoelectron Angular Distribution for Elements Z=1–100 in the Photoelectron Energy Range 1–10 KeV. *At. Data Nucl. Data Tables* **2006**, *92* (2), 245–304. https://doi.org/10.1016/j.adt.2005.12.002.

(9) Werner, W. S. M.; Smekal, W.; Powell, C. J. *Simulation of Electron Spectra for Surface Analysis (SESSA) Version 2.2.0 User s Guide*; 2021. https://doi.org/10.6028/NIST.NSRDS.100-2021.

(10) Gorgoi, M. *HIKE High Kinetic Energy Photoelectron Spectrometer*. https://www.helmholtz-berlin.de/pubbin/igama_output?modus=datei&did=147.

(11) Seah, M. P.; Smith, G. C. Quantitative AES and XPS: Determination of the Electron Spectrometer Transmission Function and the Detector Sensitivity Energy Dependencies for the Production of True Electron Emission Spectra in AES and XPS. *Surf. Interface Anal.* **1990**, *15* (12), 751–766. https://doi.org/10.1002/sia.740151208.
